# The expression of VISTA on CD4^+^ T cells is associated with poor prognosis and immune status in non-small cell lung cancer patients

**DOI:** 10.17305/bjbms.2021.6531

**Published:** 2022-02-05

**Authors:** Shengyao Ma, Liya Qin, Xinling Wang, Weiyu Wang, Jinfeng Li, Huaijie Wang, Hanyue Li, Xiaoshan Cai, Yang Yang, Meihua Qu

**Affiliations:** 1 School of Pharmacy, School of Life Science and Technology, Weifang Medical University, Weifang, China; 2 Translational Medical Center, Weifang Second People’s Hospital, The Second Affiliated Hospital of Weifang Medical University, Weifang, China; 3 Cancer Research Institute of The Fifth Medical Center, The General Hospital of the PLA, Beijing, China; 4 School of Public Health, Qingdao University, Qingdao, China

**Keywords:** VISTA, CD4^+^ T cells, non-small cell lung cancer, prognosis, immune status

## Abstract

V-domain immunoglobulin suppressor of T-cell activation (VISTA) is a novel negative immune checkpoint considered to be relevant to immunotherapy resistance and a potential target for immunotherapy. In this study, we investigated the relevance of VISTA expression on CD4^+^ T cells and clinical prognosis in non-small cell lung cancer (NSCLC) patients. Tumor tissue samples from 140 NSCLC patients were organized into a tissue microarray. The prognostic value of CD4^+^ VISTA^+^ T cells was analyzed through immunohistochemistry and multicolor fluorescence immunostaining. Fresh tumor tissue samples from 33 NSCLC patients were collected and similarly analyzed to confirm the immune state of patients with VISTA expression on CD4^+^ T cells by flow cytometry. CD4^+^ VISTA^+^ T cells were significantly more infiltrated in tumor tissues. The expression of VISTA on CD4^+^ T cells was correlated with the reduced overall survival of patients with NSCLC and presented a high rate of lymphocyte metastasis. The expression of VISTA on CD4+ T cells in tumor tissue showed low secretion of cytokines including IFN-γ, IL-2, IL-4, IL-10, IL-17, and IL-12p70. The expression of VISTA on CD4^+^ T cells could regulate tumor immunity and serve as a prognostic indicator for survival outcomes.

## INTRODUCTION

In 2020, on an international scale, there was 2.2 ­million (11.4%) new cases of lung cancer and 1.8 million (18%) deaths [1]. Non-small cell lung cancer (NSCLC) is a major type of primary lung cancer with various histological, genetic, and molecular heterogeneity [2]. Although chemotherapy, targeted therapy, and immunotherapy have been greatly developed in recent years, new immunotherapy targets are urgently required.

The interaction between tumor cells and tumor microenvironment (TME) serves a critical function in tumor occurrence, progression, metastasis, and drug resistance [3,4]. Immune cells in TME could influence the growth and evolution of tumor cells, and the cross-talking of immune cells with tumor cells significantly affects the long-term outcomes in tumor patients [5,6]. Immune checkpoints (ICs) have critical roles in modulating immune responses [7,8]. These inhibitory ICs may be an effective tumor-targeting factor. However, only 20% to 30% of NSCLC patients respond well to immune checkpoint inhibitors [9]. Therefore, the presentation of new ICs and their expression and function would possess value in cancer immunotherapy.

V-domain immunoglobulin suppressor of T-cell activation (VISTA), which was found to be overexpressed in the tumor-infiltrating lymphocytes (TILs) of NSCLC [10], might substantially regulate adaptive antitumor immunity as novel inhibitory ICs [11,12]. CD4^+^ T cells perform crucial roles in initiating, regulating, and adapting immune responses, contributing to the response mediated by B cells and CD8^+^ cytotoxic T cells [13]. The clinical significance and immune microenvironment relevant to VISTA expression on CD4^+^ T cells remain to be further explored in NSCLC. In this study, we studied the relationship between the expression of VISTA on CD4^+^ T cells and the prognosis of NSCLC patients. We tested VISTA expression on CD4^+^ T cells and identified its role in TME with the NSCLC patients’ tumor tissues. The results showed a correlation with the expression of VISTA on CD4^+^ T cells and the poor prognosis of NSCLC patients. The expression of VISTA on CD4^+^ T cells may indicate regulation of tumor immunity.

## MATERIALS AND METHODS

### Patients, and tissue microarrays

**FIGURE S1:**
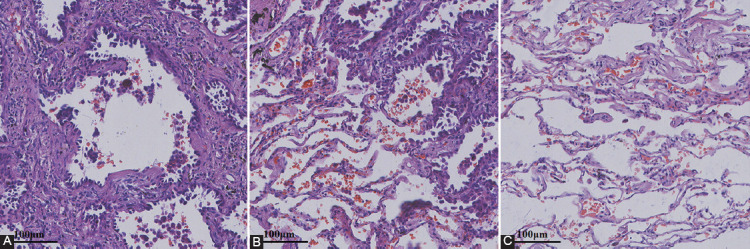
H and E staining of the tumor tissue, intermediate portion, and normal tissue. (A) Pathological characteristics of tumor tissues assessed by H and E staining. (B) H and E staining of intermediate portion. (C) H and E staining of normal tissue.

The tissue samples were collected from patients with lung adenocarcinoma subtype of NSCLC who underwent radical surgery at Weifang Second People’s Hospital from January 1 2011 to December 31 2015. Basis samples were selected randomly upon availability. The pathologist diagnosed the histological type of tumor samples through hematoxylin-eosin (H and E) staining (Supplement Figure 1). A total of 140 tumor tissue samples were organized into tissue microarray chips by Shanghai Outdo Biotech Co., LTD. The clinical and pathological information of the tissue samples were obtained from the Hospital’s information system. The overall survival (OS) was defined as the interval between the date of surgery and date of death or last follow-up. 34 patients had no prognosis and tumor, node, and metastasis (TNM) staging data.

### Immunohistochemistry (IHC)

IHC was performed on the tissue microarray slides. The tissue microarray slice was cut into 4um and baked in an oven at 60° C for 60 minutes. After dewaxing, hydrogen peroxide blocking, citric acid (BL604A, Biosharp) antigen retrieval, and goat serum (SL038, Solarbio) blocking for 30 minutes at 37° C, the slides were incubated overnight with anti-human VISTA (1:500, ab230950, Abcam) at 4° C. They were then washed by Phosphate Buffered Saline-Tween three times and incubated with Horseradish Peroxidase (HRP)-conjugated secondary antibody for 2 hours at room temperature. Thereafter, the slides were stained with 3, 30-diaminobenzidine (DAB) and counterstained with hematoxylin. The images were scanned with a Panoramic Digital Slide Scanner (3DHISTECH) and analyzed using Panoramic Viewer software (3DHISTECH).

### Multicolor fluorescence immunostaining

For multiple-color staining, the OpalTM 7-color fluorescent IHC kit was used according to the manual provided. Tissue microarrays were sequentially stained with primary antibodies and HRP-conjugated secondary antibodies. One of the four Opal reagents including Dyes Opal520, Opal620, Opal650, and Opal690 were used for staining, followed by microwave treatment and another round of staining. Anti-human CD45 (1:500, GM070129, Gene Tech), anti-human CD4 (1:500, GT219129, Gene Tech), anti-human CD8 (1:300, ab17147, Abcam), anti-human VISTA (1:500, ab230950, Abcam), anti-human CK (1:2000, ab215838, Abcam) were used as primary antibodies. Samples were visualized using the Vectra Automated Quantitative Pathology Imaging System (PerkinElmer).

### Flow cytometry

Fresh samples, including tumor tissues (n = 33) and peritumor tissues (n = 33) from NSCLC patients were collected from Weifang Second People’s Hospital. The peritumor area was defined with a radius of at least 2 cm from the tumor periphery.

Single-cell suspensions were prepared from tumor tissue. Then samples were stained with anti-human VISTA (PE, clone: MIH65, BD Pharmingen), anti-human CD45 (PC5.5, clone: J33, Beckman Coulter), anti-human CD4 (APC, clone: 13B8.2, Beckman Coulter), anti-human CD8 (APC-A750, clone: B9.11, Beckman Coulter) for 30 minutes at 4° C after being lysed with erythrocyte lysis buffer. Stained cells were washed and resuspended in phosphate-buffered saline. Flow cytometry data were analyzed by FlowJo software (BD, Bioscience).

### Cytokine determination by cytometric bead array (CBA)

Tumor tissue and peritumor tissue (100 mg) were lysed by Ultrasonic Sonicator (Virsonic 60). Cytokines IL-1β, IL-2, IL-4, IL-5, IL-6, IL-8, IL-10, IL-12p70, IL-17A, TNF-α, IFN-γ, and IFN-α, were measured in lung homogenates supernatant using the human cytokines combined detection kit (Jiangxi Cell-Gene Biotech CO., LTD) and were analyzed by software FCAP Array 3.0 (BD Biosciences) after collection of events in a flow cytometer (Beckman Coulter).

### Ethical statement

All procedures performed in studies involving human participants were in accordance with the institutional and/or national research committee’s ethical standards and with the 1964 Helsinki declaration and its later amendments or comparable ethical standards.

This study was approved by the Ethics Committee of Weifang Second People’s Hospital (approval number ky2019-004-01) and was carried out according to the guidelines of the Declaration of Helsinki.

### Statistical analysis

The survival curves were estimated by the Kaplan-Meier method. The Cox regression models were used to study the relationships between correlations and NSCLC OS. Data were analyzed using χ2 test and unpaired t-test. The statistical significance was defined as *p* < 0.05.

## RESULTS

### VISTA co-localizes with lymphocytes in tumor tissues of NSCLC patients

**FIGURE 1:**
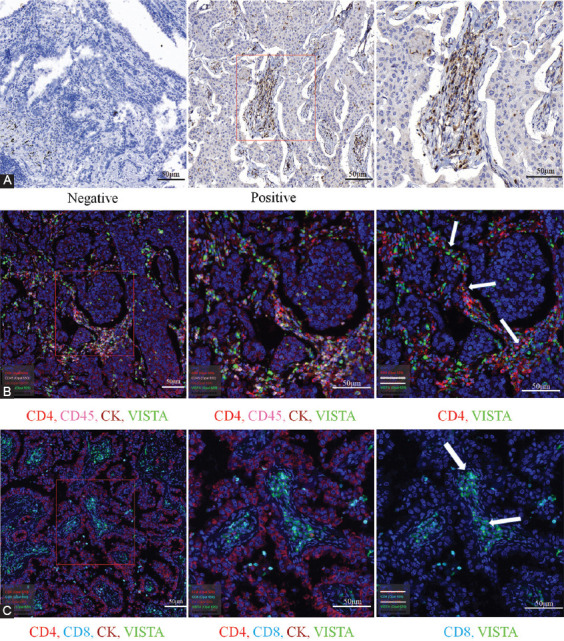
V-domain immunoglobulin suppressor of T-cell activation (VISTA) was expressed in CD45, CD4, and CD8 in non-small cell lung cancer (NSCLC). (A) Immunohistochemistry staining for VISTA in NSCLC tissues. (B) Multicolor fluorescence immunostaining for VISTA (green), CD45 (pink), CD4 (red), and CK (brown) in NSCLC tissues. (C) Multicolor fluorescence immunostaining for VISTA (green), CD8 (cyan), CD4 (red), and CK (brown) in NSCLC tissues.

The expression of VISTA in samples of NSCLC patients was identified by IHC and multicolor fluorescence immunostaining. The results showed that all 140 tumor specimens from NSCLC patients showed positive VISTA staining (representative figure shown in Figure 1A). To investigate the expression of VISTA in different immune cells, we have developed a new multiplex immunolabelling protocol with Tyramide Signal Amplification. The multicolor fluorescence immunostaining results showed that VISTA co-localized with CD45+, CD4+, and CD8+ staining lymphocytes (Figure 1B and C). These results confirmed the existence of VISTA in NSCLC lymphocytes.

### CD4^+^ VISTA^+^ T cells expression is increased in tumor tissue compared with the corresponding peritumor tissue

**FIGURE 2:**
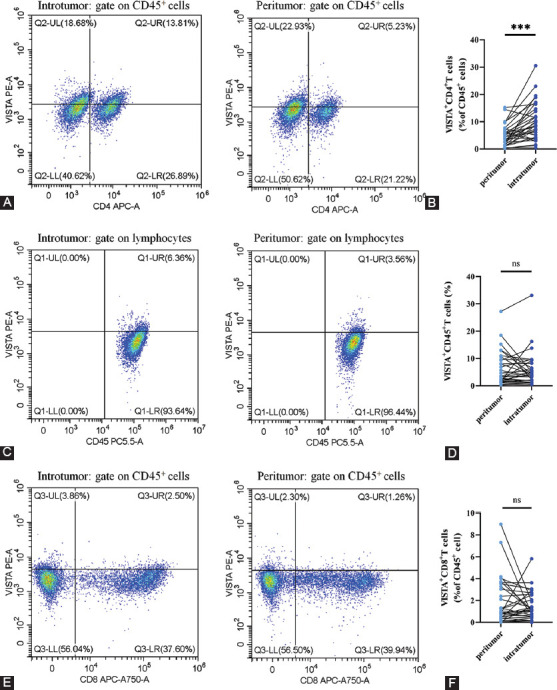
V-domain immunoglobulin suppressor of T-cell activation (VISTA) expression levels of lymphocytes were detected by flow cytometry. (A-B) Comparison of CD4^+^ VISTA^+^ T cells infiltration in tumor and peritumor tissues of patients with non-small cell lung cancer (NSCLC). (C-D) The expression levels of VISTA in CD45^+^ cells compared to peritumor tissues of patients with NSCLC. (E-F) Comparison of CD8^+^ VISTA^+^ T cell infiltration between tumor and peritumor tissues. ***p < 0.001 by unpaired t-test.

To verify the expression pattern of VISTA in fresh NSCLC tumor tissues, we analyzed the expression of VISTA in freshly collected tissues and its paired peritumor tissues by flow cytometry. The results showed that CD4+ VISTA+ T cells were significantly increased in tumor tissues compared to peritumor tissues (Figure 2A and B), while no significant difference was seen with CD45+ VISTA+ cells and CD8+ VISTA+ T cells (Figure 2C and F).

### Expression level of CD4^+^ VISTA^+^ T cells correlates with NSCLC progression and may be a prognosticator for survival outcome

**FIGURE 3:**
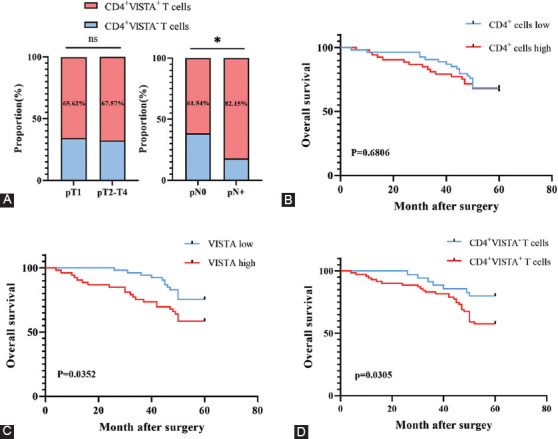
Intratumoral CD4^+^ VISTA^+^ T cells infiltration yields poor prognosis in Non-small cell lung cancer (NSCLC). (A) Proportion of patients with CD4^+^ VISTA^+/-^ T cells infiltration in pT stage and pN stage. (B) Kaplan-Meier curves for overall survival (OS) according to high/low CD4^+^ T cells infiltration in the cohort (n=106). (C) Kaplan-Meier curves for OS according to high/low VISTA in the cohort (n=106). (D) Kaplan-Meier curves for OS according to CD4^+^ VISTA^+/-^ T cells infiltration in the cohort (n=106). Log-rank test was performed for Kaplan-Meier curves. *P<0.05 by χ2 test.

We analyzed the expression of VISTA in 106 NSCLC patients undergoing surgical treatment at different TNM stages. The patient characteristics and tumor pathological features are presented in Table 1. The ratio of patients with CD4+ VISTA+ T cells infiltration increased with advanced pathology Node (pN) staging, but no difference was found in pathology Tumor (pT) staging (Figure 3A). Due to the comparatively small number of cases with metastasis, analysis was not performed in this stage.

**TABLE 1:**
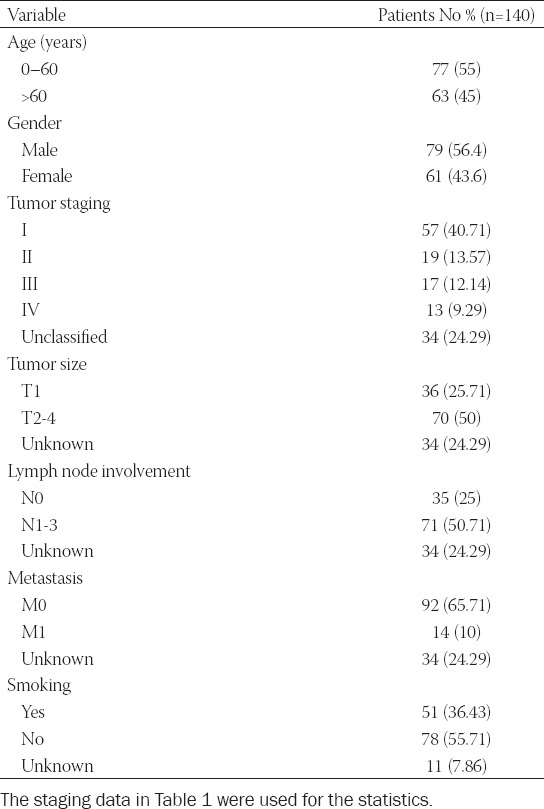
Patient characteristics

The prognostic ability of the expression of VISTA on CD4+ T cells was explored next. Among all NSCLC cases with a follow-up of at least 5 years, the OS showed no difference with CD4+ T cell group (*p* = 0.6806, Figure 3B). The high expression of VISTA was related to decreased OS of patients with NSCLC (*p* = 0.0352, Figure 3C). Next, we sought to explore whether the expression of VISTA on CD4+ T cells affects the prognosis of NSCLC patients. The CD4+ VISTA+ T cells group possessed inferior OS than that of the CD4+ VISTA-T cells group (*p* = 0.0305, Figure 3D). These results indicated that the expression of VISTA on CD4+ T cells correlates with NSCLC progression and may be a factor in predicting the survival of NSCLC patients. Univariate Cox regression analysis indicated that node stage (*p* < 0.001), metastasis stage (*p* = 0.05), VISTA expression abundance (*p* = 0.02), and the expression of VISTA on CD4+ T cells (*p* = 0.038) were significantly correlated with OS. Multivariate Cox regression analysis showed that node stage (*p* < 0.001) and VISTA expression abundance (*p* = 0.041) were two independent prognostic factors for OS, while the metastasis stage and the expression of VISTA on CD4+ T cells were not (Table 2).

**TABLE 2:**
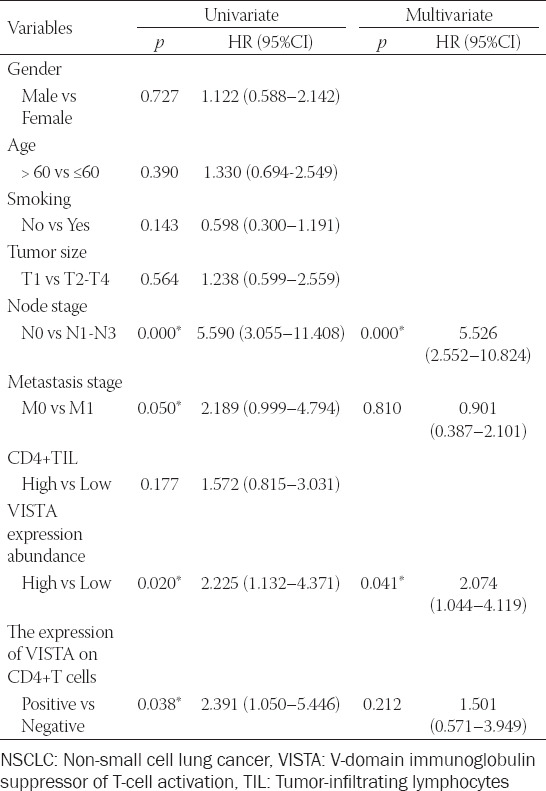
Univariate and multivariate analyses of prognostic parameters in 106 NSCLC patients by Cox-regression analysis

### The expression of VISTA on CD4+ T cells may affect immunity in patients with NSCLC

**FIGURE 4:**
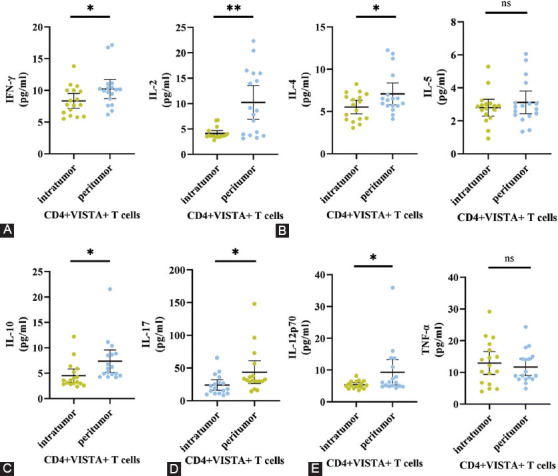
The expression of V-domain immunoglobulin suppressor of T-cell activation (VISTA) on CD4+ T cells may affect immunity in patients with non-small cell lung cancer (NSCLC). (A) Expression of Th1 cytokines (IFN-γ, n = 17; IL-2, n = 17) in the tumor tissue compared with the peritumor tissue (B) The contents of Th2 cytokines (IL-4, n = 17; IL-5, n = 17) in the tumor tissue compared with the peritumor tissue. (C) Expression of Tregs cytokine (IL-10, n = 17) in the tumor tissue compared with the peritumor tissue. (D) The production of Th17 cytokine (IL-17, n = 17) in the tumor tissue compared with the peritumor tissue. (E) The level of macrophage cytokines (IL-12p70, n = 17; TNF-α, n = 17) in the tumor tissue compared with the peritumor tissue. *p < 0.05, **p < 0.01 by unpaired t-test.

**FIGURE S2:**
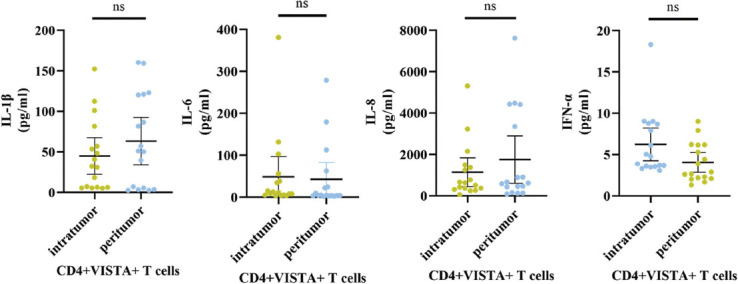
The expression of CD4+ T cells may be correlated with the immune status of patients with Non-small cell lung cancer. There was no significant difference in other cytokines.

Levels of Th1, Th2, regulatory T cells (Tregs), and Th17 and macrophage cytokine secretion in NSCLC patients with VISTA expression on CD4+ T cells were subsequently investigated. We evaluated the cytokine levels of tumor specimens and peritumor specimens in patients with NSCLC. The results showed that the cytokine level in TME of patients with VISTA expression on CD4+ T cells was lower than that in matched adjacent peritumor tissues. Th1 cytokines (IFN-γ, IL-2), Th2 cytokine (IL-4), Tregs cytokine (IL-10), and Th17 cytokine (IL-17) in TME were lower than matched adjacent peritumor tissues (Figure 4A-D). In addition, the level of macrophage cytokine (IL-12p70) in the tumor tissue was significantly reduced compared with the peritumor tissue (Figure 4E). IL-1β, IL-6, IL-8, and IFN-α showed no significant differences (Supplement Figure 2). Together with the results in Figure 4, these data demonstrate that the expression of VISTA on CD4+ T cells should inhibit the function of immune system within the tumor tissue compared with matched adjacent peritumor tissue.

## DISCUSSION

Immune cells play an important role in the progression of NSCLC. The function of immune cells in the tumor is primarily divided into two categories: promoting or inhibiting tumor progression. In a study by Catacchio et al. on the association between immune cells and prognosis in lung cancer, it was reported that M2 macrophages, immature dendritic cells, and Treg cells were found to be associated with poor prognosis [14]. Next, we tentatively explored immune cells that were associated with poor prognosis and the mechanism of functional and mechanical barriers. M2 macrophages secreted immunosuppressive cytokines, chemokines, and growth factors to inhibit T-cell activation and promote tumor progression [15,16]. Treg cells also can secrete immunosuppressive cytokines and express cytotoxic T lymphocytes cell antigen-4 (CTLA-4) to impair antitumor immune responses [17,18]. A study showed that oxidative stress can induce apoptosis of Treg cells and release a large amount of adenosine triphosphate, which is metabolized into adenosine through the high expression of CD39 and CD73 in Treg cells. Adenosine binds to A2A receptor (A2AR) to inhibit effector T cells [19]. In lung cancer, the presence of immature dendritic cells led to poor prognosis due to overexpression of programmed cell death-L1 (PD-L1) and programmed cell death-L2 (PD-L2) to inhibit T cell proliferation and activation [20,21]. Myeloid-derived suppressor cells (MDSCs) have been reported to be highly involved in the multistep development of tumorigenesis. MDSC can highly express indoleamine 2mine3-dioxygenase (IDO) which can degrade 1-tryptophan to N-formylcanine to inhibit T-cell activation and promote CD4+ T cells to differentiate into Treg cells [22]. MDSCs could promote tumor progression through several other mechanisms, such as upregulating PD-L1, secreting immunosuppressive cytokines and growth factors, secreting high levels of vascular endothelial growth factor, and basic fibroblast growth factor (bFGF) [23-25]. These cells contribute to poor progression mainly by inhibiting T-cell activation and proliferation. It is also important to note that the density and activation status of tumor-infiltrating T cells may be critical indicators of prognosis for NSCLC. A study showed that more TILs infiltrated the tumor environment in NSCLC when normally CD4+ T cells account for a large percentage [26]. CD4+ T cells display an antitumor immunity profile by enhancing the antineoplastic effect at the tumor site, preventing activation-induced cell death, and giving priority to the generation of immune memory cells by cytotoxic T-lymphocytes [27]. CD4+ T cells play an important role in antitumor immunity. However, the potential role of CD4+ T cells in the prognosis of NSCLC is still unclear.

VISTA was identified as a potent inhibitor of T-cell proliferation and activation, which are crucial in immune suppression [11,28-30]. VISTA overexpressed in TILs was studied in various cancers, including melanoma [31], gastric cancer [32], and colorectal cancer [33]. Co-localization was prominent between VISTA and TILs by multicolor fluorescence immunostaining results. Our results showed that CD4+ VISTA+ T cells were significantly increased in fresh NSCLC tumor tissues compared with peritumor tissues. CD4+ VISTA+ T cell expression may correlate with tumor progression in NSCLC.

A study by Wu et al. showed that VISTA expression on CD8+ T cells may predict the OS of oral squamous cell carcinoma [34]. However, the association between VISTA expression on CD4+ T cells and survival was not clearly determined. Our results demonstrated that the expression of VISTA on CD4+ T cells was correlated with lymph node metastasis and poor prognosis in NSCLC patients. Although the expression of VISTA on CD4+ T cells has been implicated in tumor progression in a recent study, it should be cautiously assumed based on the small patient numbers that the expression of VISTA on CD4+ T cells may be a novel clinical prognostic parameter for NSCLC.

We examined the levels of cytokines in the TME by CBA. Cytokine concentration between tumor tissue and matched adjacent peritumor tissue was significantly different. The tumor tissue of patients with VISTA expression on CD4+ T cells had a lower level of cytokines. Decreased global levels of cytokines in TME showed that intratumor immune cells may be more inactive than peritumor immune cells. The result suggests that the expression of VISTA on CD4+ T cells may affect immunity in patients with NSCLC.

## CONCLUSION

In summary, the results of this study indicated that the expression of VISTA on CD4+ T cells was related to poor prognosis in NSCLC patients and may be used as a prognostic factor for adverse NSCLC outcomes. The expression of VISTA on CD4+ T cells correlated with the immune status of the NSCLC patients. These findings indicated that VISTA might be considered a potential target for NSCLC immunotherapy.
